# Supplementary material for: Rebels with a cause? How norm violations shape dominance, prestige, and influence granting
Source: PLoS One. 2023 Nov 21;18(11):e0294019. doi: 10.1371/journal.pone.0294019 (PMC10662731; doi:10.1371/journal.pone.0294019)
Supplement: S1 File — Additional methodological details, data, and analyses. (DOCX) [file pone.0294019.s001.docx]

# Supporting Information

# pertaining to

# Rebels with a cause:

# How norm violations shape dominance, prestige, and influence granting

In this supporting information document we present additional details, data, and analyses that are not central to understanding or evaluating the arguments and results presented in the main text but that may nevertheless be of interest to some readers.

**Study 1**

The implicit association test (IAT) employed in Study 1 was administered to Dutch participants, using Dutch target words. S1 Table shows the original Dutch words used in the study together with the English translations.

**Study 2**

**Additional measures**

Besides the dependent variables of interest and the measure of perceived power (which was included for the purpose of replication), we included a number of additional measures in Study 2 for exploratory purposes. These included measures of inferred descriptive and injunctive norms pertaining to car parking, the perceived appropriateness of the protagonist’s behavior, and participants’ own tendencies toward illegal parking. These measures did not yield insights pertinent to the present investigation.

**Study 3**

**Additional analyses**

In the main text we report the results of one-way ANOVAs, which allowed for the most targeted test of our hypotheses. Here we present the results of an alternative analytical procedure that is less suitable for testing our specific hypotheses but that yields some additional insight, namely 2 x 2 ANOVAs in which the effects of abidance by versus violation of community norms and abidance by versus violation of group norms on dominance, prestige, and influence granting can be tested separately and in interaction.

**Dominance**

2 x 2 ANOVA yielded a significant main effect of the community norm manipulation on perceptions of dominance, *F*(1, 370) = 60.60, *p* <.001, η_p_^2^ = .14, such that a protagonist who violated the global community norm was perceived as more dominant (*M* = 4.58, *SD* = 1.04) than a protagonist who abided by the community norm (*M* = 3.81, *SD* = 0.84). We also observed a significant, albeit weaker, main effect of the local group norm manipulation on perceptions of dominance, *F*(1, 370) = 4.88, *p* = .028, η_p_^2^ = .01, such that a protagonist who violated the local group norm was also perceived as being more dominant (*M* = 4.32, *SD* = 0.99) than a protagonist who abided by the local group norm (*M* = 4.08, *SD* = 1.04). The interaction between group norm and community norm violation versus abidance was non-significant, *F*(1, 370) = 0.32, *p* = .573, η_p_^2^ = .00. These patterns are in line with Hypothesis 1. However, they also reveal an additional nuance, in that dominance perceptions were shaped more strongly by violation of the community norm than by violation of the group norm.

**Prestige**

2 x 2 ANOVA revealed a significant main effect of the group norm manipulation on perceptions of prestige, *F*(1, 370) = 92.10, *p* < .001, η_p_^2^ = .96, such that the protagonist was perceived as more prestigious when abiding by (*M* = 4.96, *SD* = 0.86) rather than violating the local group norm (*M* = 4.08, *SD* = .93). There was no significant main effect of the community norm manipulation on perceptions of prestige, *F*(1, 370) = 0.15, *p* = .702, η_p_^2^ = .00 (*M_violate_* = 4.50, *SD_violate_* = 1.01; (*M_abide_* = 4.56, *SD_abide_* = 0.98), and no interaction, *F*(1, 370) = 2.31, *p* = .129, η_p_^2^ = .01. Consistent with our theoretical arguments, these results indicate that group norm violations had a stronger impact on prestige than did community norm violations.

**Influence granting: Intersubjective leadership endorsement**

2 x 2 ANOVA yielded a significant main effect of the group norm manipulation on intersubjective leadership endorsement, *F*(1, 370) = 262.46, *p* < .001, η_p_^2^ = .42, such that protagonists who abided by the group norm were more likely to be endorsed as leaders (*M* = 5.33, *SD* = 1.11) than those who violated the group norm (*M* = 3.18, *SD* = 1.48). There was no significant main effect of community norm violation on leadership endorsement, *F*(1, 370) = 0.212, *p* = .645, η_p_^2^ = .00 (*M_violation_* = 4.28, *SD_violation_* = 1.83; *M_abidance_* = 4.28, *SD_abidance_* = 1.53), but we did observe a significant interaction, *F*(1, 370) = 13.43, *p* = < .001, η_p_^2^ = .04 (see Table 2 in the main text for *M*s and *SD*s). Simple effects analyses indicated that the effect of the protagonist’s group norm abidance versus violation on leadership endorsement was somewhat stronger when the protagonist violated (*t*[370] = 2.95, *p* = .003, *d* = 0.51) rather than abided by (*t*[370] = 2.24, *p* = .026, *d* = 0.29) the community norm. As a result, leadership endorsement was highest when the protagonist abided by the group norm while violating the community norm.

**Discussion**

These additional analyses indicate that global community norm violations had a more potent impact on perceptions of dominance than local group norm violations. A possible explanation for this finding is that participants inferred that violating global community norms could have more severe consequences for the transgressor than violating local community norms, because the global community (in this case: the university) is a more powerful entity than the local group (in this case: the fraternity/sorority). Viewed in this light, the observed difference in the magnitude of the effects of global community norm violations versus local group norm violations is consistent with the theoretical argument underlying our hypothesis that norm violations fuel perceptions of dominance, namely that norm violators signal that they can withstand any pushback their behavior may entail. If the potential pushback of violating community norms is larger than that of violating group norms, then violators of community norms may come across as especially dominant. The additional analyses further revealed that prestige was influenced by local group norm violations, but not by global community norm violations. This finding is in keeping with the theoretical notion that prestige tracks individuals’ value and potential benefits to the group, more so than their value to the broader community (1). Finally, the additional analyses indicate that the combination of local group norm abidance and global community norm violation elicited the greatest leadership endorsement, more than overall abidance. This may be because community norm violators signal that they are willing to incur a risk in order to honor the group’s norms, which may make them even more attractive as leaders in the eyes of fellow group members (2).

**Additional measures**

Besides the dependent variables of interest, we included a number of additional measures in Study 3 for exploratory purposes. These included measures of perceived warmth and competence, volitional capacity, trustworthiness, and bravery of the target, perceived risk of the target’s behavior, and the extent to which the target appeared to identify with the fraternity/sorority and university. We also included a number of personality measures, including honesty, humility, trait dominance, and moral identity. Finally, we asked participants whether they had been part of a fraternity or sorority, and what the color(s) of their school and sorority/fraternity were. These measures did not yield pertinent insights.

**Study 4**

**Alternative operationalization of global community norms**

As reported in the main text, we included an alternative operationalization of global community norm violation in the form of the violation of the norms of students outside of the peer group (see S2 Table). Using this alternative operationalization did not change the results and conclusions. When community norms were operationalized not as school norms but as norms of students outside of the peer group, popular students were again reported to have violated community norms to a greater extent (*M =* 46.45*, SD =* 27.60) than control students (*M =* 33.16*, SD =* 27.67), *t*(54) = 3.02, *p* = .004, *d* = 0.41.

**Additional measures**

Besides the dependent variables of interest, Study 4 included a number of additional measures that were not pertinent to the present investigation. These included questions about the participant’s relationship with the two students they reflected on (whether they belonged to the same group and whether they still had contact with either student), and participants’ ratings of both students in terms of warmth, competence, liking, admiration, and malicious envy. These measures did not yield additional insights.

## **Study 5**

**Treatment of missing values**

In Study 5 there were missing values on the behavioral measure of assignment of leadership tasks. To enable mediation analyses involving the full set of observations on the independent variable and mediator, we imputed missing values on the dependent variable using a number of different simulation approaches: expansion, stochastic regression imputation, and predictive mean matching.

**Expansion**

In the expansion method, missing responses are imputed with their corresponding predicted regression weights from regressing the behavioral measure of assignment of leadership tasks on condition and the mediator(s). Adding random error to these imputed values ensures that the associated *p* value for the effect of prestige on the assignment of leadership tasks is not inflated. We chose this random error such that the resulting *p* value for the effect of prestige on assignment of leadership tasks would be similar, but slightly higher, compared to the *p* value obtained with the original data. In other words, we opted for a conservative test of the indirect effect.

**Stochastic imputation**

Stochastic regression imputation follows the same steps as the expansion algorithm, except that estimates are not chosen based on a desired *p* value (3).

**Predictive mean matching**

Expansion and **s**tochastic regression imputation may produce values outside the range of possible values (3). Although this does not represent a problem for the analysis of the current data as we are interested in the relationship between variables rather than the absolute values of any specific variable, we also estimated the coefficients using predictive mean matching, which overcomes the problem of implausible values by choosing values of the dependent variable that are similar to the ones observed for similar predictors (3).

These different imputation approaches returned highly similar estimates. In the paper we report the results of the expansion method. For the sake of comprehensiveness, we report the estimates obtained from all three approaches here. S3 Table presents the results of models including both dominance and prestige as candidate mediators and S4 Table presents the results of models including only prestige. Path coefficients yielded by stochastic imputation and predictive mean matching are presented in S1 and S2 Figs, respectively.

**S1 Fig. Multiple mediation model testing the effect of conflict between local group norms and global community norms on assignment of leadership tasks via prestige and dominance after imputing missing values on the dependent variable via stochastic imputation (Study 5).**

**S2 Fig. Multiple mediation model testing the effect of conflict between local group norms and global community norms on assignment of leadership tasks via prestige and dominance after imputing missing values on the dependent variable via predictive mean matching (Study 5).**

**Additional measures**

Besides the dependent variables of interest, Study 5 involved a number of additional measures that are not pertinent to the present investigation. These included measures of warmth and competence, expected negative emotional reactions (anger, disgust, and contempt) from fellow team members, and the extent to which the target appeared to identify with the team and the company. These measures did not yield relevant insights.

## **References**

1. Henrich J, Gil-White FJ. The evolution of prestige: freely conferred deference as a mechanism for enhancing the benefits of cultural transmission. Evol Hum Behav [Internet]. 2001 May;22(3):165–96. Available from: http://dx.doi.org/10.1016/s1090-5138(00)00071-4

2. van Kleef GA, Heerdink MW, Cheshin A, Stamkou E, Wanders F, Koning LF, et al. No guts, no glory? How risk-taking shapes dominance, prestige, and leadership endorsement. J Appl Psychol [Internet]. 2021 Nov;106(11):1673–94. Available from: http://dx.doi.org/10.1037/apl0000868

3. van Buuren S, Groothuis-Oudshoorn K. mice: Multivariate Imputation by Chained Equations inR. J Stat Softw [Internet]. 2011;45(3). Available from: http://dx.doi.org/10.18637/jss.v045.i03
