# Supplementary material for: Rebels with a cause? How norm violations shape dominance, prestige, and influence granting
Source: PLoS One. 2023 Nov 21;18(11):e0294019. doi: 10.1371/journal.pone.0294019 (PMC10662731; doi:10.1371/journal.pone.0294019)
Supplement: S1 Table — (DOCX) [file pone.0294019.s002.docx]

**S1 Table. Original Dutch target words employed in the Implicit Association Test (IAT) together with English translations (Study 1).**

| Norm abidance | |  | Norm violation | |
| --- | --- | --- | --- | --- |
| *Dutch original* | *English translation* |  | *Dutch original* | *English translation* |
| gehoorzaam | obedient |  | tegendraads | unruly |
| opvolgen | follow |  | schenden | violate |
| naleven | comply |  | rebels | rebellious |
| meegaand | accommodating |  | overtreden | transgress |
| braaf | well-behaved |  | stout | naughty |
|  |  |  |  |  |
| Dominance | |  | Prestige | |
| *Dutch original* | *English translation* |  | *Dutch original* | *English translation* |
| overheersend | domineering |  | bewonderd | admired |
| bazig | bossy |  | status | status |
| dwingend | forceful |  | prestigieus | prestigious |
| dominantie | dominance |  | respectabel | respected |
| autoritair | authoritarian |  | eervol | honorable |
