# Supplementary material for: Rebels with a cause? How norm violations shape dominance, prestige, and influence granting
Source: PLoS One. 2023 Nov 21;18(11):e0294019. doi: 10.1371/journal.pone.0294019 (PMC10662731; doi:10.1371/journal.pone.0294019)
Supplement: S2 Table — (DOCX) [file pone.0294019.s003.docx]

**S2 Table. The extent to which a popular and a typical (control) student violated norms of teachers, other students, and peers (Study 3)*.*** Means within a row with a different subscript differ at *p* < .05. All measurers were recorded on a 100mm visual analogue scale (0 = *never*, 100 = *always*).

| Norm Violation | Popular | Control |
| --- | --- | --- |
| Teachers’ norms | 46.00 (26.18) _a_ | 21.51 (23.36) _b_ |
| Other students’ norms | 46.45 (27.60) _a_ | 33.16 (27.67) _b_ |
| Peers’ norms | 18.73 (16.17) _a_ | 20.47 (22.20) _a_ |
