# Supplementary material for: Rebels with a cause? How norm violations shape dominance, prestige, and influence granting
Source: PLoS One. 2023 Nov 21;18(11):e0294019. doi: 10.1371/journal.pone.0294019 (PMC10662731; doi:10.1371/journal.pone.0294019)
Supplement: S3 Table — (DOCX) [file pone.0294019.s004.docx]

**S3 Table. Indirect effects of condition on the assignment of leadership tasks via prestige and dominance as estimated by three different imputation methods (Study 5).**

|  | Estimated Path Coefficients | | | | | |
| --- | --- | --- | --- | --- | --- | --- |
| Method | a_1_ (Prestige) | b_1_ (Prestige) | a_1_×b_1_ (Prestige) | a_2_ (Dominance) | b_2_ (Dominance) | a_2_×b_2_ (Dominance) |
|  |  |  |  |  |  |  |
| Expansion | *b* = 0.31, *t*(174) = 2.07*, p* = .040 | *b* = 0.37, *t*(172) = 3.96*, p < .*001 | *b* = 0.12, 95% CI [0.008, 0.261] | *b* = 0.18, *t*(174) = 1.17, *p* = .244 | *b* = 0.13, *t*(172) = 1.40, *p* = .163 | *b =* 0.02, 95% CI  [-0.024, 0.095] |
|  |  |  |  |  |  |  |
| Stochastic imputation | *b* = 0.31, *t*(174) = 2.07*, p* = .040 | *b* = 0.39, *t*(172) = 5.10*, p < .*001 | *b* = 0.12, 95% CI [0.008, 0.261] | *b* = 0.18, *t*(174) = 1.17, *p* = .244 | *b* = 0.06, *t*(172) = 0.87, *p* = .383 | *b =* 0.01, 95% CI  [-0.026, 0.068] |
|  |  |  |  |  |  |  |
| Predictive mean matching | *b* = 0.31, *t*(174) = 2.07*, p* = .040 | *b* = 0.34, *t*(172) = 4.61*, p < .*001 | *b* = 0.11, 95% CI [0.010, 0.218] | *b* = 0.18, *t*(174) = 1.17, *p* = .244 | *b* = 0.07, *t*(172) = 0.98, *p* = .329 | *b =* 0.01, 95% CI  [-0.022, 0.067] |
