# Supplementary material for: Rebels with a cause? How norm violations shape dominance, prestige, and influence granting
Source: PLoS One. 2023 Nov 21;18(11):e0294019. doi: 10.1371/journal.pone.0294019 (PMC10662731; doi:10.1371/journal.pone.0294019)
Supplement: S4 Table — (DOCX) [file pone.0294019.s005.docx]

**S4 Table. Indirect effects of condition on the assignment of leadership tasks via prestige as estimated by three different imputation methods (Study 5).**

|  | Estimated Path Coefficients | | |
| --- | --- | --- | --- |
| Method | a | b | a×b |
|  |  |  |  |
| Expansion | *b* = 0.31, *t*(174) = 2.07*, p* = .040 | *b* = 0.37, *t*(173) = 3.96*, p < .*001 | *b* = 0.12, 95% CI [0.008, 0.250] |
|  |  |  |  |
| Stochastic imputation | *b* = 0.31, *t*(174) = 2.07*, p* = .040 | *b* = 0.38, *t*(173) = 5.02*, p < .*001 | *b* = 0.12, 95% CI [0.010, 0.249] |
|  |  |  |  |
| Predictive mean matching | *b* = 0.31, *t*(174) = 2.07*, p* = .040 | *b* = 0.33, *t*(173) = 4.51*, p < .*001 | *b* = 0.10, 95% CI [0.005, 0.210] |
