# Supplementary figures and images for: Rebels with a cause? How norm violations shape dominance, prestige, and influence granting
Source: PLoS One. 2023 Nov 21;18(11):e0294019. doi: 10.1371/journal.pone.0294019 (PMC10662731; doi:10.1371/journal.pone.0294019)

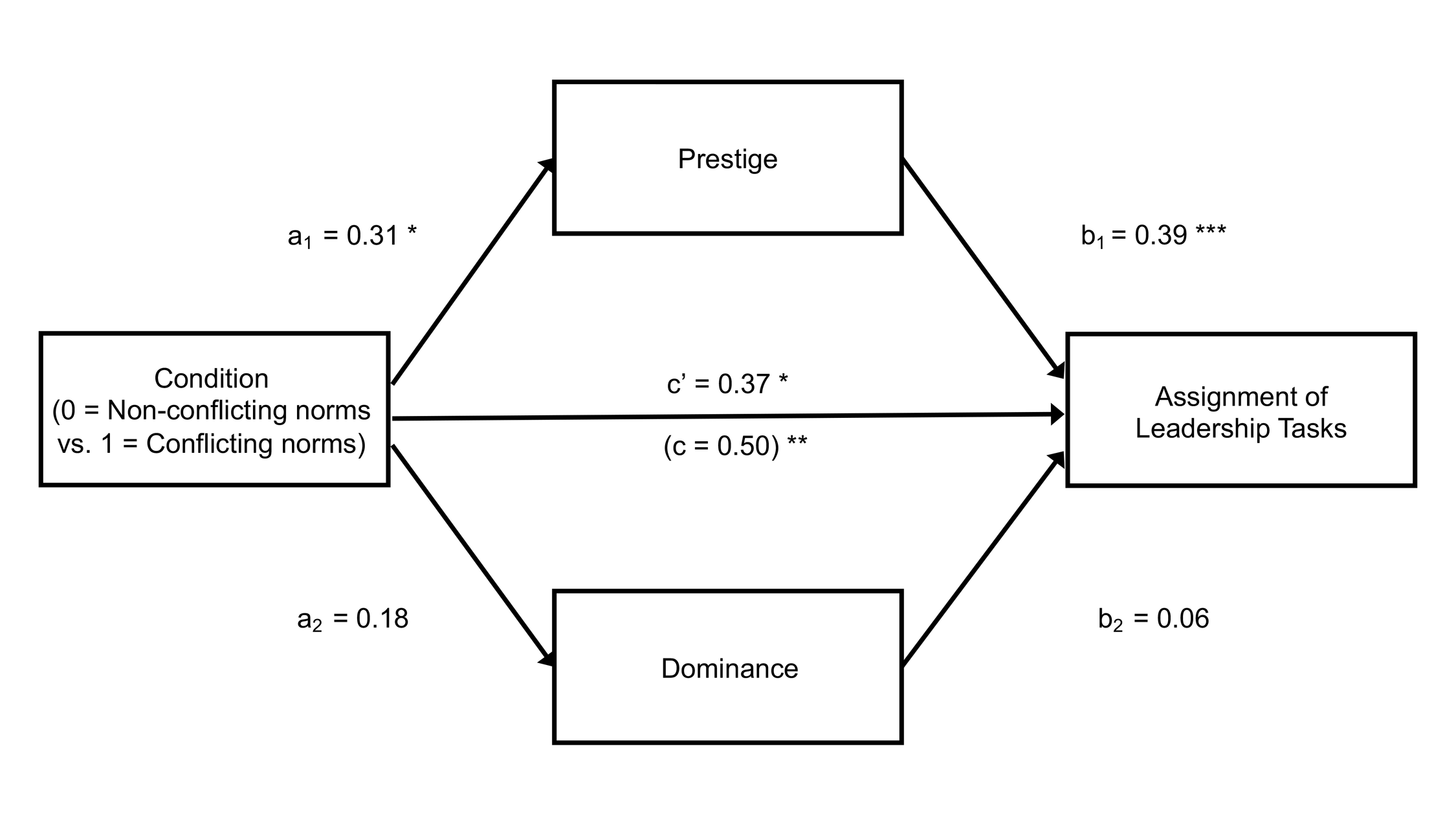

Supplement: S1 Fig — (TIF) [file pone.0294019.s006.tif]

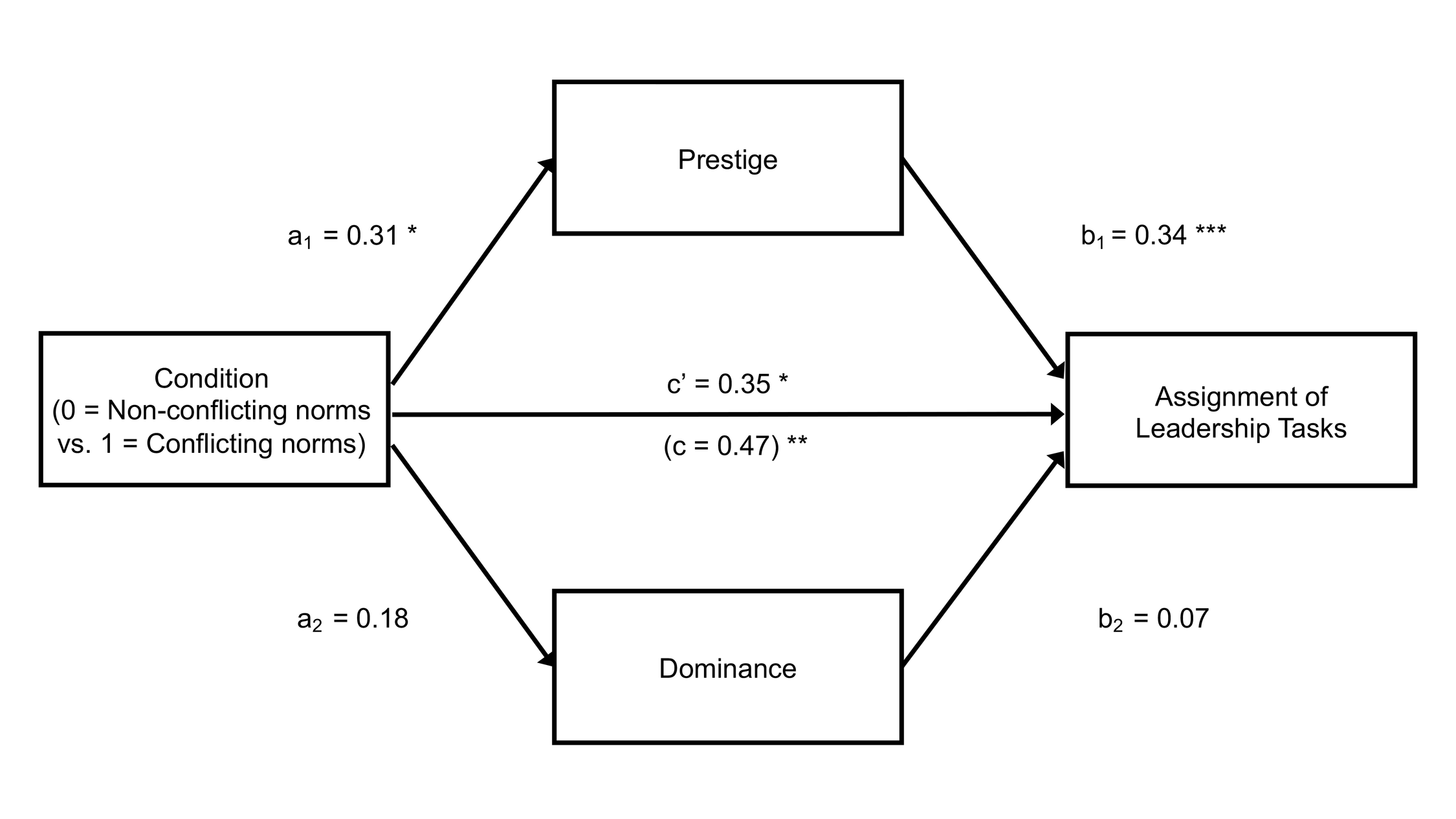

Supplement: S2 Fig — (TIF) [file pone.0294019.s007.tif]
