# Supplementary material for: Rebels with a cause? How norm violations shape dominance, prestige, and influence granting
Source: PLoS One. 2023 Nov 21;18(11):e0294019. doi: 10.1371/journal.pone.0294019 (PMC10662731; doi:10.1371/journal.pone.0294019)
Supplement: Attachment — Submitted filename: Rebels with a Cause (Response to Reviewers) R1.docx [file pone.0294019.s008.docx]

**Response Letter Pertaining to PONE-D-23-23034**

Rebels with a Cause?

How Norm Violations Shape Dominance, Prestige, and Influence Granting

**Editor’s Comments**

*Thank you for submitting your manuscript to PLOS ONE. After careful consideration, we feel that it has merit but does not fully meet PLOS ONE’s publication criteria as it currently stands. Therefore, we invite you to submit a revised version of the manuscript that addresses the points raised during the review process.*

Thank you for giving us the opportunity to revise and resubmit our paper for further consideration. Below we indicate how we have dealt with each of your and the reviewers’ responses.

*Please submit your revised manuscript by Oct 05 2023 11:59PM. If you will need more time than this to complete your revisions, please reply to this message or contact the journal office at* [*plosone@plos.org*](mailto:plosone@plos.org)*. When you're ready to submit your revision, log on to* [*https://www.editorialmanager.com/pone/*](https://www.editorialmanager.com/pone/) *and select the 'Submissions Needing Revision' folder to locate your manuscript file.*

*Please include the following items when submitting your revised manuscript:*

- *A rebuttal letter that responds to each point raised by the academic editor and reviewer(s). You should upload this letter as a separate file labeled 'Response to Reviewers'.*
- *A marked-up copy of your manuscript that highlights changes made to the original version. You should upload this as a separate file labeled 'Revised Manuscript with Track Changes'.*
- *An unmarked version of your revised paper without tracked changes. You should upload this as a separate file labeled 'Manuscript'.*

*If you would like to make changes to your financial disclosure, please include your updated statement in your cover letter. Guidelines for resubmitting your figure files are available below the reviewer comments at the end of this letter.*

We look forward to receiving your revised manuscript.

*Journal Requirements:*

*When submitting your revision, we need you to address these additional requirements.*

*1. Please ensure that your manuscript meets PLOS ONE's style requirements, including those for file naming. The PLOS ONE style templates can be found at* 
[*https://journals.plos.org/plosone/s/file?id=wjVg/PLOSOne_formatting_sample_main_body.pdf*](https://journals.plos.org/plosone/s/file?id=wjVg/PLOSOne_formatting_sample_main_body.pdf) *and* 
[*https://journals.plos.org/plosone/s/file?id=ba62/PLOSOne_formatting_sample_title_authors_affiliations.pdf*](https://journals.plos.org/plosone/s/file?id=ba62/PLOSOne_formatting_sample_title_authors_affiliations.pdf)

We have worked through the paper to ensure that the reporting fits the journal’s style requirements. The main changes we made in this regard concern the formatting of the title page, headers, table and figure captions, and references. We applied similar changes to the Supporting Information document.

*2. We note that you have stated that you will provide repository information for your data at acceptance. Should your manuscript be accepted for publication, we will hold it until you provide the relevant accession numbers or DOIs necessary to access your data. If you wish to make changes to your Data Availability statement, please describe these changes in your cover letter and we will update your Data Availability statement to reflect the information you provide.*

Thank you for clarifying this process. We have meanwhile uploaded our data on OSF (<https://osf.io/f42wq/?view_only=2924f496672d4a30a13d17653177b11e>). We have also added this link to the manuscript on p. 13.

*3. Please include your full ethics statement in the ‘Methods’ section of your manuscript file. In your statement, please include the full name of the IRB or ethics committee who approved or waived your study, as well as whether or not you obtained informed written or verbal consent. If consent was waived for your study, please include this information in your statement as well.*

We have now added the full name of the ethics review board in the methods sections of all studies. Please note that we already stated explicitly in each study that we obtained written consent. In case this is not the type of information you are looking for, we would very much appreciate your guidance as to what to add further.

*4. Please include captions for your Supporting Information files at the end of your manuscript, and update any in-text citations to match accordingly. Please see our Supporting Information guidelines for more information:* [*http://journals.plos.org/plosone/s/supporting-information*](http://journals.plos.org/plosone/s/supporting-information)*.*

We have added captions at the end of the main text, prior to the reference list (see p. 65).

*5. Please review your reference list to ensure that it is complete and correct. If you have cited papers that have been retracted, please include the rationale for doing so in the manuscript text, or remove these references and replace them with relevant current references. Any changes to the reference list should be mentioned in the rebuttal letter that accompanies your revised manuscript. If you need to cite a retracted article, indicate the article’s retracted status in the References list and also include a citation and full reference for the retraction notice.*

We have double checked our reference list to make sure it is complete and accurate. To the best of our knowledge, we are not citing any research that has been retracted.

*Please read all reviewers' comments and revise your paper carefully.* 

We have carefully considered each of the reviewers’ thoughtful comments, and we provide a point-by-point response below.

**Reviewers' Ratings**

*1. Is the manuscript technically sound, and do the data support the conclusions?

The manuscript must describe a technically sound piece of scientific research with data that supports the conclusions. Experiments must have been conducted rigorously, with appropriate controls, replication, and sample sizes. The conclusions must be drawn appropriately based on the data presented.*

*Reviewer #1: Yes*

*Reviewer #2: Yes*

We are pleased that both reviewers deemed the manuscript technically sound and the data supportive of the conclusions.

*2. Has the statistical analysis been performed appropriately and rigorously?*

Reviewer #1: Yes

Reviewer #2: Yes

We appreciate the reviewers’ favorable responses to the appropriateness and rigor of the statistical analyses.

*3. Have the authors made all data underlying the findings in their manuscript fully available?

The* [*PLOS Data policy*](https://eur04.safelinks.protection.outlook.com/?url=http%3A%2F%2Fwww.plosone.org%2Fstatic%2Fpolicies.action%23sharing&data=05%7C01%7Cg.a.vankleef%40uva.nl%7C1565a2745b9a4d3a363908dba2084e09%7Ca0f1cacd618c4403b94576fb3d6874e5%7C0%7C0%7C638281930789884116%7CUnknown%7CTWFpbGZsb3d8eyJWIjoiMC4wLjAwMDAiLCJQIjoiV2luMzIiLCJBTiI6Ik1haWwiLCJXVCI6Mn0%3D%7C3000%7C%7C%7C&sdata=MLkmNYn8hnRob1lP%2BTe4eCL9%2F29amEbhRqDjszfgKYw%3D&reserved=0) *requires authors to make all data underlying the findings described in their manuscript fully available without restriction, with rare exception (please refer to the Data Availability Statement in the manuscript PDF file). The data should be provided as part of the manuscript or its supporting information, or deposited to a public repository. For example, in addition to summary statistics, the data points behind means, medians and variance measures should be available. If there are restrictions on publicly sharing data—e.g. participant privacy or use of data from a third party—those must be specified.*

*Reviewer #1: No*

*Reviewer #2: No*

We have meanwhile uploaded the data of all five studies on a public repository (see <https://osf.io/f42wq/?view_only=2924f496672d4a30a13d17653177b11e>). This link is also provided in the paper on p. 13.

*4. Is the manuscript presented in an intelligible fashion and written in standard English?

PLOS ONE does not copyedit accepted manuscripts, so the language in submitted articles must be clear, correct, and unambiguous. Any typographical or grammatical errors should be corrected at revision, so please note any specific errors here.*

*Reviewer #1: Yes*

*Reviewer #2: Yes*

We are glad that the reviewers found the writing of the manuscript to be clear, correct, and unambiguous.

**Reviewer 1’s Comments**

*Thank you for the opportunity to review "Rebels with a Cause? How Norm Violations Shape Dominance, Prestige, and Influence Granting". In this manuscript, the authors examine the effect of norm violations on status conferral. The manuscript is well written, the experiments are well designed, and the manuscript concludes with insightful predictions for future research. My concerns are minimal, but I would appreciate the authors providing clarity in their response letter on the following points.*

Thank you for your positive feedback on our work, it is much appreciated.

*The author’s argument is that individuals gain prestige when they violate global norms in order to comply with local norms. And the theoretical underpinning is that by complying with the local norm at the expense of the global norm, the violator is engaging is a costly act of support for the group, and as a result garners prestige. The authors provide support for this by showing that members of the local group (or participants answering from the perspective of someone from the local group) rate the violator as more prestigious in such contexts.*

*However, might not members of the global community have a very different perspective of such violators? I doubt they would also grant prestige to such violators. So, is the argument actually more nuanced: individuals gain prestige from their local group and lose prestige from their global group when they violate global norms in order to comply with local norms? And on net wouldn’t the violator actually lose prestige given that the global group consists of more people than the local group? I suppose prestige from one’s local group is particularly beneficial, so much so that it sufficiently offsets the decline in prestige amongst the broader community, making such behaviour adaptive.*

Thank you for this insightful comment. We agree that the favorable effects of global norm violations on prestige are likely to be limited in that violators would gain prestige in the eyes of their fellow group members but not necessarily in the eyes of others. Indeed, this possibility is consistent with our theoretical argument that social norms are contextualized and that prestige has evolved as a mechanism to highlight individuals who possess attributes that generate benefits for the group. It is possible, as you noted, that the enhanced prestige in the eyes of fellow group members goes hand in hand with reduced prestige in the eyes of others. Although we have no data speaking directly to this possibility, Studies 1 and 2 do suggest that norm violations generally undermine prestige. We appreciate your thoughtful suggestion that violators would thus lose prestige in the eyes of the majority while gaining prestige in the eyes of a minority that is particularly relevant for them, thereby rendering the net outcome of the behavior adaptive. We have worked through the Introduction of the manuscript to make sure we properly contextualize and nuance our argument, and we have expanded our discussion of this issue in the General Discussion (see p. 62).

*Relatedly, is this really about a comparison of global versus local, or is it just a comparison of allegiance to one group as opposed to another group? For example, wouldn’t the same effect be observed if one chose to attend their football club’s match, thereby forgoing attendance at their rugby club’s match. The violator would gain prestige with their football club while losing prestige with their rugby club. Is the local versus global comparison necessary for this effect to occur? If the global versus local comparison is necessary, does the scope of comparison matter? For example, imagine that nested within a local group was a micro group. Would breaching the micro group’s norms for the sake of the local group’s norms result in a gain or loss of prestige? And what would each outcome imply for the authors’ theory?*

This is a very interesting line of thought. We share your intuition that similar effects may occur in comparisons between different groups. The reason we focused our investigation on groups nested within larger communities is that the implications of violations in such social constellations are particularly impactful as they affect large groups of people (e.g., members of society at large as opposed to members of the local rugby team) and thereby undermine the normative systems that hold societies together. Moreover, we believe that any conflict between different sets of norms is likely to be more salient in nested group constellations than in non-nested group constellations as it is not possible to “escape” the norms of the global community in the former case because the local group is a part of it. That said, we believe the theoretical extension to different constellations of groups is very interesting as it potentially extends the scope of our theoretical model. We have added a paragraph to the General Discussion in which we speculate about this possibility (see pp. 62-63).

*The authors demonstrate in Study 5 that what is key for garnering prestige is that the compliance with the local norm requires the violation of the global norm. In other words, the violator needs to be a ‘rebel with a cause’ rather than a rebel without one. If that is the case, I am curious to know whether the authors see the results from Study 4 fitting with this argument. Were the popular students in Study 4 violating the teacher’s norms in order to comply with the norms of their peers? Or were they simply violating the teacher’s norms in order to be rebellious (without a cause)? I realise that this was a recall study which doesn’t allow for the same level of precision on such matters, but I would appreciate the authors to speculate on this point as it does seem central to their theorizing.*

This is an interesting question. You are correct in noting that the recall methodology we used in this study does not enable strong inferences in this regard. The pattern we observed – that popular students more frequently violated global rather than local norms – is compatible with the possibility that these students violated global norms in order to be able to follow local norms, but clearly this is not direct proof. What would be needed in order to be able to draw a more definitive conclusion from these data is insight in the degree to which global and local norms were at odds with each other. We suggest that the likelihood of this being the case may be relatively high in the context of high school classes, in which students often have different sets of norms than teachers. We have added a paragraph to the discussion of Study 4 in which we offer this suggestion, while noting explicitly that the data of this study do not allow for firm conclusions in this regard (see p. 42).

*Typo on bottom of Page 24: “…rated the protagonist as les[s] prestigious…”*

Thank you for catching this typo – we have corrected it.

**Reviewer 2’s Comments**

*I commend the authors on producing a well-written manuscript built on sound empirical studies. I believe this has the potential to be an important contribution across a range of disciplines.

Overview:

This study focuses on understanding the relationship between norm violations and influence within social groups. The authors explore how norm violators either gain or lose influence via variation in their perceived dominance and prestige. They found that norm violations are linked to perceptions of dominance, while norm abidance is associated with prestige. The authors conducted a series of integrated studies, including experimental designs, demonstrating that when local group norms and global community norms conflict, individuals who violate global norms but adhere to local norms are perceived as both dominant and prestigious, leading to greater influence and endorsement as leaders. This research sheds light on the complex interplay of norms, influence, and leadership emergence within social groups and variation within and between social groups.

The authors are correct in their assertion that much of the literature on norm violations and influence overlooks the importance of social context and nested and overlapping social groups. The authors make an important contribution in emphasizing this point and incorporating it into their empirical frameworks.

I find the methods appropriate and results convincing.*

Thank you for your favorable evaluation of our research and for your thoughtful pointers for further improvement.

*My main critics are 1) I would prefer if the authors would have better conformed to open science practices, including preregistering predictions and analyses. Perhaps I missed it, but I also did not see that data and code are openly available. I think the data and code are only available upon publication. Therefore, I cannot evaluate “behind the scenes”. I realize, however, that the data were collected several years ago. The authors could be more straight forward about when or why these practices were not followed and if any elements of “researcher degrees of freedom” influenced processes or decisions. And, 2) I would prefer to see more discussion about the limitations of the sample, the particularities of the samples, and generalizability, and how the sample characteristics constrain what these results can tell us about “human” cognition or social behavior.*

We appreciate your comment. Indeed, most of the studies were conducted several years ago. That said, we made every effort to comply with open science practices. As noted in our response to the Editor, we have meanwhile made our data publicly available (see <https://osf.io/f42wq/?view_only=2924f496672d4a30a13d17653177b11e>). Moreover, we are fully transparent in our reporting, providing complete information about deleted cases (which were few) and additional measures. While the first four studies have not been pre-registered, they are all theoretically grounded, build on previous research, and are hypothesis-driven. Our main hypotheses that people generally associate norm violations with dominance (H1) and norm abidance with prestige (H2) were formulated prior to Study 1 and successively tested in Studies 1 and 2. Our hypotheses 3 to 5 were developed prior to Study 3 and successively tested in Studies 3 and 4. This procedure left no room for decisions to deviate from these hypotheses. Based on the results of Studies 1 to 4, we developed hypotheses 6 to 8, which we tested in a pre-registered Study 5 (see p. 44).

Regarding particularities and limitations of the samples, we have added a paragraph to the General Discussion in which we discuss sample characteristics and associated limitations of our five studies (see p. 59). We acknowledge that student samples (which we used in Studies 1, 2, and 4) are rather demographically homogeneous, which may limit generalizability. We also note that our studies involving general-population samples (which we used in Studies 3 and 5) produced compatible findings, which enhances our confidence in the generalizability of our conclusions.

*There is another relevant idea I did not see authors discuss. I’m thinking about, when high status, prestigious leaders are able to violate certain norms because of their high status and prestige. A common example is ethnography includes high status big men in horticultural societies being able to strategically violate marriage norms in finding/taking a wife. In such cases, the community is aware that they have violated a norm and the community has available mechanisms to punish the leader for the norm violation. But, because of their history of prosocial investments and good leadership, these sorts of global norm violations may be overlooked. There could be other more local norm violations as well. I suppose, a core point of this idea is that naturalistic individual behavior often does not exist in a one-shot interaction (and this was probably rarely the case over human evolutionary history). Therefore, a reputation and history of prestigious prosociality may afford an individual the right to violate particular norms in certain contexts.*

*See:

Lewis, H. S. (1974). Leaders and followers: Some anthropological perspectives. Addison-Wesley.

Boehm, C. (1999). Hierarchy in the forest: The evolution of egalitarian behavior. Harvard University Press Cambridge, MA.

Chagnon, N. A. (1968). Yaomamö, the fierce people. Holt, Rinehart and Winston.*

Indeed, classic research on “idiosyncrasy credits” (Hollander, 1958) and more recent work on “transgression credit” and “innovation credit” (Abrams et al., 2008; 2013) suggests that individuals who have developed a positive reputation receive greater leeway to violate norms. In addition, there is evidence that individuals who violate norms in ways that benefit others (i.e., prosocial norm violations) are preferentially afforded power (Van Kleef et al., 2012). You seem to point to a reciprocal process in which individuals violate a norm for the good of their group, leading to greater prestige and leadership, which in turn gives them greater freedom to violate norms. We find this possibility very interesting, but we feel that a discussion along these lines would have to be very tentative as our data do not allow us to draw conclusions about cyclical processes. We therefore chose not to discuss this issue for now, so as to maintain focus. However, if you feel that a discussion along these lines would really benefit the paper, we will be happy to reconsider.

Abrams, D., Randsley de Moura, G., Marques, J. M., & Hutchison, P. (2008). Innovation credit: When can leaders oppose their group's norms?. Journal of Personality and Social Psychology, 95(3), 662-678. doi:10.1037/0022-3514.95.3.662

Abrams, D., Randsley de Moura, G., & Travaglino, G. A. (2013). A double standard when group members behave badly: Transgression credit to ingroup leaders. Journal of Personality and Social Psychology, 105(5), 799-815. doi: <https://doi.org/10.1037/a0033600>

Hollander, E. P. (1958). Conformity, status, and idiosyncrasy credit. *Psychological Review, 65*(2), 117–127. [https://doi.org/10.1037/h0042501](https://psycnet.apa.org/doi/10.1037/h0042501)

*Other comments:

Line 149: It may be worth mentioning that the degree to which, and/or when dominance and prestige are more or less distinct has been challenged on both theoretical and empirical grounds.

See:

Von Rueden, C. (2014). The roots and fruits of social status in small-scale human societies. In J. T. Cheng, J. L. Tracy, & C. Anderson (Eds.), The psychology of social status (pp. 179–200). Springer.

von Rueden, C., Gurven, M., Kaplan, H., & Stieglitz, J. (2014). Leadership in an Egalitarian Society. Hum Nat, 25(4), 538–566.* [*https://doi.org/10.1007/s12110-014-9213-4*](https://doi.org/10.1007/s12110-014-9213-4) *Chapais, B. (2015). Competence and the Evolutionary Origins of Status and Power in Humans. Hum Nat, 26, 161–183.* [*https://doi.org/10.1007/s12110-015-9227-6*](https://doi.org/10.1007/s12110-015-9227-6) *Jiménez, Á. V., & Mesoudi, A. (2019). Prestige-biased social learning: Current evidence and outstanding questions. Palgrave Communications, 5(1), 20.* [*https://doi.org/10.1057/s41599-019-0228-7*](https://doi.org/10.1057/s41599-019-0228-7) *Garfield, Z. H., & Hagen, E. H. (2020). Investigating evolutionary models of leadership among recently settled Ethiopian hunter-gatherers. Special Issue on Evolution and Biology of Leadership, 31(2), 101290.* [*https://doi.org/10.1016/j.leaqua.2019.03.005*](https://doi.org/10.1016/j.leaqua.2019.03.005)

Thank you for pointing us to this work. As we understand most of these papers, the distinction between dominance and prestige (or coercion and collaboration; Chapais, 2015) may not always be as clear as suggested in the theoretical and empirical work by Henrich and Gil-White (2001) and Cheng and colleagues (2013), because traits or characteristics that are associated with dominance (e.g., physical strength) are sometimes also associated with expertise relevant for the survival of the group (e.g., conflict resolution; e.g., Garfield & Hagen, 2020; Von Rueden et al., 2014). Whereas we appreciate this nuance (and acknowledge it in the paper on p. 60), our conclusion from these papers is that even though prestige and dominance might go together, there is still evidence for both prestige-related influence and dominance-related influence. Interestingly, Garfield and Hagen (2020) actually found that women leaders were only perceived as higher on prestige, not dominance. Similarly, Von Rueden et al. (2011) found that prestigious but not dominant men marry wives who give birth at earlier ages and experience lower pre-adult mortality. This work again underlines that these processes are distinct to a certain degree (if dominance and prestige could not be separated, these findings would not have been possible), and that even though dominance and prestige might go together, they have different correlates and differentially shape relevant outcomes.

We would further like to reiterate here that the distinction between dominance and prestige has been widely supported, both theoretically and empirically. Theoretically, dominance and prestige capture notably different processes. Whereas dominance involves behaviors such as intimidation, threat, and force, prestige reflects the degree to which individuals make their knowledge, skills, and abilities available for the benefit of the group and for others to learn from (Henrich & Gil-White, 2001). Put differently, dominance is associated with the forceful claiming of influence, whereas prestige is associated with the voluntary conferral of influence by fellow group members out of respect. Empirically, a large body of work has demonstrated that dominance and prestige are distinct and viable routes to influence (e.g., Cheng et al., 2013; Halevy et al., 2012; Maner & Case, 2016), which can also be observed in real-life settings in organizations, politics, and society at large (e.g., Blake, 2022; Garfield & Hagen, 2020; Kakkar & Sivanathan, 2017; Redhead et al., 2012; Van Kleef et al., 2021; Von Rueden et al., 2011). Nevertheless, as noted, we now mention the caveat that dominance and prestige are not always as clearly separable in the limitations section (p. 60).

*Line 159-162: An important nuance, however, includes that followers may prefer dominant leaders and preferentially select them as leaders in the context of cooperative activities, given their superior abilities to deter free riders. Therefore, dominance can be an effective leadership strategy/suit of characteristics for promoting followership.

See:

von Rueden, C. R. (2022). Unmaking egalitarianism: Comparing sources of political change in an Amazonian society. Evolution and Human Behavior.* [*https://doi.org/10.1016/j.evolhumbehav.2022.09.001*](https://doi.org/10.1016/j.evolhumbehav.2022.09.001)

We have made a minor edit to our formulation to better reflect this nuance (see p. 7).

*Line 165-166: This trend also appears to be widespread across human societies.

See:

Lewis, H. S. (1974). Leaders and followers: Some anthropological perspectives. Addison-Wesley.

Garfield, Z. H., Hubbard, H., Robert, & Hagen, E. H. (2019). Evolutionary models of leadership: Tests and synthesis. Human Nature, 30(1), 23–58.* [*https://doi.org/10.1007/s12110-019-09338-4*](https://doi.org/10.1007/s12110-019-09338-4)

We have added a reference to Garfield et al. to further support our argument here (see p. 8).

*Line 168: The authors are correct that the social learning theory of the evolution of prestige has been largely influential, however, it may be worth noting there are alternative theoretical frameworks developed in recent years, particularly those focused on benefit generation and cost infliction capacities.

See:

Von Rueden, C. (2014). The roots and fruits of social status in small-scale human societies. In J. T. Cheng, J. L. Tracy, & C. Anderson (Eds.), The psychology of social status (pp. 179–200). Springer.

Pietraszewski, D. (2019). The evolution of leadership: Leadership and followership as a solution to the problem of creating and executing successful coordination and cooperation enterprises. The Leadership Quarterly.* [*https://doi.org/10.1016/j.leaqua.2019.05.006*](https://doi.org/10.1016/j.leaqua.2019.05.006) *Hagen, E. H., & Garfield, Z. (2019). Leadership and prestige, mothering, sexual selection, and encephalization: The computational services model [Preprint]. Open Science Framework.* [*https://doi.org/10.31219/osf.io/9bcdk*](https://doi.org/10.31219/osf.io/9bcdk) *Garfield, Z. H., Syme, K. L., & Hagen, E. H. (2020).* *Universal and variable leadership dimensions across human societies. Evolution and Human Behavior, 41(5), 397–414.* [*https://doi.org/10.1016/j.evolhumbehav.2020.07.012*](https://doi.org/10.1016/j.evolhumbehav.2020.07.012) *Durkee, P. K., Lukaszewski, A. W., & Buss, D. M. (2020). Psychological foundations of human status allocation. Proceedings of the National Academy of Sciences, 117(35), 21235–21241.* [*https://doi.org/10.1073/pnas.2006148117*](https://doi.org/10.1073/pnas.2006148117)

Thank you for pointing us to these papers. We have read this work very carefully and came to the conclusion that incorporating it in our manuscript would make our argument unnecessarily complex and unfocused. We therefore decided against discussing these perspectives explicitly. That said, we believe our phrasing is sufficiently broad to encapsulate the benefit generation perspective, even if it is not discussed explicitly, as the word benefit plays a central part in our argument (“such voluntary granting of influence is more commonly observed in connection with prestige, which is thought to have evolved as a psychological adaptation to highlight individuals who possess attributes and dispositions that generate benefits for the group”; p. 7).

*Line 199-201: Is this necessarily true? People may violate norms unintentionally. Or, they may have some prior expectation on the likelihood their violations will be met with sanctions. And based on other contextual factors (which the authors review well) they may “compute” some set of cost-benefit analyses on the probability of backlash and the severity of any potential backlash they may be subjected to.*

We agree that the knowing and intentional violation of norms in particular signals willingness and ability to withstand backlash (see Bellezza et al., 2014). And indeed, prior expectations and cost-benefit analyses may play a role in an individual’s decision to violate a norm, but these cognitions are difficult to observe by others. We have changed the formulation of our argument as follows to make it explicit that it pertains to norm violations that are perceived as intentional: “People who appear to knowingly and intentionally violate norms signal that they are willing and able to withstand such backlash” (see p. 8).

*Line 200-203: I would also suggest the embodied capital/wealth inequality model is important here, i.e., individuals well endowed with certain forms of capital, e.g., social, material, or embodied capital, may be able to withstand or recuperate from certain forms of backlash.

See:

Von Rueden, C. (2014). The roots and fruits of social status in small-scale human societies. In J. T. Cheng, J. L. Tracy, & C. Anderson (Eds.), The psychology of social status (pp. 179–200). Springer.

Mattison, S. M., Smith, E. A., Shenk, M. K., & Cochrane, E. E. (2016). The evolution of inequality. Evolutionary Anthropology: Issues, News, and Reviews, 25, 184–199.

Lancaster, J. B., & Kaplan, H. S. (2010). Embodied Capital and Extra-somatic Wealth in Human Evolution and Human History. In M. P. Muehlenbein (Ed.), Human Evolutionary Biology (pp. 439–456). New York: Cambridge University Press.*

We agree that individuals who possess certain forms of capital may also be able to withstand backlash. However, the point we wish to make here is that norm violators signal dominance, because dominant individuals have the capacity to withstand backlash and prevail in confrontations. Although interesting, the possibility that certain forms of capital similarly shield people against backlash is not pertinent to this argument. We therefore decided not to incorporate this literature to maintain focus.

*Introduction, generally: The authors points about local and group norm norms and nested social groups is compelling and important. For recent and consistent cross-cultural work highlighting the importance of socio-ecology in shaping norm violations see:

Garfield, Z. H., Ringen, E. J., Buckner, W., Medupe, D., Wrangham, R. W., & Glowacki, L. (2023). Norm violations and punishments across human societies. Evolutionary Human Sciences, 5, e11.* [*https://doi.org/10.1017/ehs.2023.7*](https://doi.org/10.1017/ehs.2023.7)

Thank you for pointing us to this work. Although we find this perspective very interesting, our argument is not about how the socio-ecology shapes punishment after norm violations, but about how norm violations shape perceptions of dominance and prestige and subsequent influence granting. That said, it is certainly possibly that the processes we uncovered are shaped by socio-ecological variables, and we believe this constitutes an exciting avenue for future research. We have added a sentence along these lines in our future directions section on p. 64.

*Line 298: Were the predictions and/or methods preregistered? If not, can the authors justify or discuss this decision?*

Please see our response to your first main comment. Studies 1 to 4 were conducted several years ago when preregistration of predictors and methods was not yet common research practice. However, based on theory and (our and others’) prior research we developed our hypotheses and methods prior to conducting and analyzing these studies and no adjustments were made during the research process. The last study in the package, Study 5, was pre-registered.

*General comment: The framework the authors have developed (and used in their predictions) seems to rely on the assumption of distinctiveness between dominance and prestige, which is fair. But the authors also explain well that nested groups and norms overlap and can contradict. The same principle applies to the conceptions and representations of dominance and prestige, as the authors have defined them. For example, if "aggression, coercion, and intimidation" are seen as locally valued "skills, expertise, or abilities", would the expression and embodiment of these qualities be defined as dominance or prestige? This seems to be important concerning the hypotheses to be tested.*

Thank you for raising this point. Indeed, what affords prestige should depend on the specific attributes and dispositions that generate benefits for the group. In our discussion section we now mention the possibility that prestige and dominance can overlap in social contexts where dominant behaviors are seen as beneficial to the group (see p. 60).

*Study 1: Perhaps I missed it, but were participants given any explanation or definitions of dominance and prestige?*

Participants were not provided with definitions. We used five different words for each category. For norm abidance, we used obedient, follow, comply, accommodating, and well-behaved; for norm violation, we used unruly, violate, rebellious, transgress, and naughty; for dominance, we used domineering, bossy, forceful, dominance, and authoritarian; and for prestige, we used admired, status, prestigious, respected, and honorable. We conducted a pretest with a larger set of words and selected those words for each category that were most consistently paired with the intended category by our pretest participants. This procedure is described on p. 16, where we have now made it explicit that this procedure ensured that the selected words were understood as intended.

*General discussion:

The authors have done well to discuss their samples and participants. However, I feel they could/should discuss the limitations and generalizability in greater detail. Particularly in drawing on theoretical frameworks rooted in evolutionary theory (i.e., the dominance-prestige framework as developed by the cited authors). There are a few minor instances of fairly broad language in the author’s discussions. For example, “across the board” (Line 1192) and “individuals” (Line 1196). I realize that a diligent reader will not make the mistake of over generalizing the author’s interpretations. But, as a safeguard and in respect with current best practices, I suggest discussing the potential limits to generalizability, based on what is known about the participant sample. The cultural tightness-looseness or WEIRD people frameworks might be useful. That is, given the samples of students, etc., what can the authors say about their results and generalizability, in a bit more detail.

For detailed discussions of external validity and ways to discuss in a comprehensive fashion see:

Findley, M. G., Kikuta, K., & Denly, M. (2021). External Validity. Annual Review of Political Science, 24(1), 365–393.* [*https://doi.org/10.1146/annurev-polisci-041719-102556*](https://doi.org/10.1146/annurev-polisci-041719-102556)

We have carefully checked our language in the General Discussion to ensure we do not make undue claims about generalizability. For instance, we replaced “across the board” with “in our samples” and we now write “individuals appear to more readily amass prestige…” to further nuance our conclusions (see p. 54). In addition, we have added a paragraph discussing possible constraints on generalizability imposed by our student samples, which we suggest are partly compensated by the use of general-population samples in two of our studies (see p. 59). Note that we also discuss the role of culture as a possible constraint on the generalizability of our conclusions (see pp. 63-64).

*While revising your submission, please upload your figure files to the Preflight Analysis and Conversion Engine (PACE) digital diagnostic tool,* [*https://pacev2.apexcovantage.com/*](https://eur04.safelinks.protection.outlook.com/?url=https%3A%2F%2Fpacev2.apexcovantage.com%2F&data=05%7C01%7Cg.a.vankleef%40uva.nl%7C1565a2745b9a4d3a363908dba2084e09%7Ca0f1cacd618c4403b94576fb3d6874e5%7C0%7C0%7C638281930789884116%7CUnknown%7CTWFpbGZsb3d8eyJWIjoiMC4wLjAwMDAiLCJQIjoiV2luMzIiLCJBTiI6Ik1haWwiLCJXVCI6Mn0%3D%7C3000%7C%7C%7C&sdata=EzZhR1N3iCaLqAd5AaWqwE0QzbLvZz%2BfD3OHdL93y2M%3D&reserved=0)*. PACE helps ensure that figures meet PLOS requirements. To use PACE, you must first register as a user. Registration is free. Then, login and navigate to the UPLOAD tab, where you will find detailed instructions on how to use the tool. If you encounter any issues or have any questions when using PACE, please email PLOS at* [*figures@plos.org*](mailto:figures@plos.org)*. Please note that Supporting Information files do not need this step.*

We have run our figures through PACE to optimize them and uploaded the new versions onto the submission portal.
